# The 2009-2014 economic crisis and deaths by suicide in Portugal: time series analysis

**DOI:** 10.1093/eurpub/ckac129.263

**Published:** 2022-10-25

**Authors:** V Pinheiro, C Caetano, S Pereira da Silva, B Nunes

**Affiliations:** 1 CINTESIS, Centre for Health Technology and Services Research, Porto, Portugal; 2 Public Health Unit, ACES Arco Ribeirinho, ARS LVT, Lisbon, Portugal; 3 National Health Institute Dr. Ricardo Jorge, Lisbon, Portugal

## Abstract

**Introduction:**

Population health, including mental health, is influenced by its socioeconomic context. After the 2008 global economic crisis, studies found contradicting results: some showed an increased risk for self-harm and suicidal behavior, while others found the opposite association. To the best of our knowledge, there is no research in Portugal on the subject. Thus, our aim was to estimate the impact of the Portuguese economic crisis of 2009-2014 on the death rate by suicide and self-inflicted injury in Portugal.

**Methods:**

A retrospective ecological study with an interrupted time series analysis of deaths by suicide and self-inflicted injury (data from the National Statistics Institute) in mainland Portugal, in 2003-2014, was performed. Resident population data was also retrieved from the National Statistics Institute. Binomial negative generalized linear models were used to compare rates and trends before (2003-2008) and during (2009-2014) the economic crisis. All rates were stratified and adjusted for seasonality.

**Results:**

The economic crisis was associated with 13% a step increase in the death rate due to suicide and self-inflicted injury, with unemployment playing a significant mediating role, being negatively associated to the outcome. Differences between groups exist, with males, working-age groups and the North and Centre regions being the most impacted, globally.

**Conclusions:**

Economic downturns pose risks for suicidal behavior. Unemployment may play a role in this association. Employment protection schemes can prevent this impact, so urgent action is needed to prevent economic crisis leading to additional suicides, especially in the context of the COVID-19 pandemic and the economic crisis it caused.

**Key messages:**

• The Portuguese economic crisis of 2009-2014 was associated with an increased death rate due to suicide, especially in males, working-age groups and the North and Centre regions.

• Unemployment may play a role in this association, and active labour market programmes can prevent the negative impacts of economic crisis leading to additional suicides.

